# How consistent are recent neonatal resuscitation guidelines?

**DOI:** 10.1016/j.resplu.2026.101314

**Published:** 2026-04-03

**Authors:** Ilari Kuitunen, Peter G. Davis

**Affiliations:** a University of Eastern Finland, Institute of Clinical Medicine and Department of Pediatrics, Kuopio, Finland; b Kuopio University Hospital, Department of Pediatrics, Kuopio, Finland; c University of Melbourne, Melbourne, Australia; d Royal Women’s Hospital, Melbourne, Australia

## Abstract

**Background:**

Approximately 10% of neonates require respiratory or cardiovascular support at birth, making evidence-based neonatal resuscitation guidelines critical to clinical practice.

**Methods:**

This narrative review compares the most recent neonatal resuscitation guidelines published by the American Heart Association/American Academy of Pediatrics (AHA/AAP), the Australian and New Zealand Committee on Resuscitation (ANZCOR), and the European Resuscitation Council (ERC), all derived from the 2025 International Liaison Committee on Resuscitation (ILCOR) recommendations.

**Results:**

While the three guidelines share common foundations, some notable differences exist in the method of grading and transparency of assessment of evidence certainty, and strength of recommendations. Key comparisons include umbilical cord management, airway and ventilation strategies, oxygen use, vascular access, and pharmacologic interventions during resuscitation. All guidelines recommend delayed cord clamping in vigorous infants, discourage routine airway suctioning for meconium-stained fluid, and support ventilation within 60 s of delivery when indicated. However, recommendations diverge regarding intact cord stabilization, cord milking in extremely preterm infants, initial oxygen concentrations, ventilation parameters, and the use of devices such as video laryngoscopes, laryngeal mask airways, and high-flow nasal cannula.

**Conclusion:**

Although the AHA/AAP, ANZCOR, and ERC neonatal resuscitation guidelines are all derived from the same ILCOR evidence summaries, important differences exist in their structure, transparency, and specific clinical recommendations. In part, differences in recommendations are due to a paucity of evidence around specific interventions. Standardized methods of reporting certainty of evidence and strength of recommendations may help clinicians deliver consistent, effective delivery room care.

## Introduction

Globally, neonatal mortality rates are declining.^1^ The neonatal mortality rate in term infants has reached a nadir of 0.4 per 1000 liveborn neonates.^2^, ^3^ A growing number of neonates born at 22 and 23 weeks’ gestation are now offered treatment and many survive.^4^, ^5^ Improved antenatal and neonatal care have contributed to the improved survival of both term and preterm neonates.^6^

Approximately 10% of neonates receive assistance in the first minutes of life, most commonly supplemental oxygen (8% of all neonates) and continuous positive airway pressure (7% of all neonates).^7^ Among late preterm and term neonates, delivery of positive pressure ventilation (PPV) is reported to vary between 3% and 6%.^8^, ^9^ The provision of initial care is provided in accordance with local and national guidelines, mostly derived from recommendations formulated by the International Liaison Committee on Resuscitation (ILCOR) which regularly updates International Consensus on Cardiopulmonary Resuscitation and Emergency Cardiovascular Care Science With Treatment Recommendations. The latest update was published in 2025.^10^ Neonatal resuscitation is an active field for research and new studies are published regularly, meaning guidelines struggle to keep up with evolving evidence.^11^

The updates of neonatal resuscitation guidelines by American Heart Association and American Academy of Pediatrics (AHA/AAP), Australian Resuscitation Council (ANZCOR), and European Resuscitation Council (ERC) were recently published.^12^, ^13^, ^14^ This review compares these guidelines and discusses the evidence underpinning them. In conducting this narrative review, we highlighted the topics we felt were of most interest to clinicians, particularly those where new evidence was available or where marked differences in recommendations were observed. We therefore have not addressed topics where substantial evidence has been available for many years and on which guidelines agree, like thermal management.

## Characteristics of the included guidelines

The three guidelines included were all based on the most recent ILCOR recommendations (Table 1). There were key differences in the structure of the guidelines. The AHA/AAP and ERC guidelines were published as peer reviewed journal articles, whereas the ANZCOR guidelines were published online under 10 subheadings (Table 1). The AHA/AAP recommendations followed the methodology of AHA guideline panels.^15^ They included assessment of the class (strength) of recommendation and the level (quality) of evidence. The quality of evidence was rated from Level A to Level C (Table 1).^15^ ANZCOR provided an assessment of the certainty (quality) of evidence using the GRADE approach i.e. providing a ranking from very low to high.^16^, ^17^, ^18^ The GRADE approach was also used to provide a strength of recommendation, either strong or weak. If evidence was insufficient to provide a recommendation, the ANZCOR guideline made good practice statements, similar to ILCOR.^19^ The ERC guidelines differ in that formal assessments of evidence certainty and recommendation strength are not presented within the guideline document. Instead, the ERC recommendations are derived from the ILCOR evidence evaluations and are presented in a concise format intended to support rapid decision-making in emergency settings.^20^

**Table 1:** Characteristics of the AHA/AAP 2025, ANZCOR, and ERC 2025 neonatal resuscitation guidelines.

	**AHA/AAP 2025**	**ANZCOR**	**ERC 2025**
Definitions	Neonate <28 days	Neonate <28 days	Neonate <28 days
Gestational age focus	Not specified	Not specified	>24 + 6 gestational weeks
Based on	ILCOR reviews and recommendations	ILCOR reviews and recommendations	ILCOR reviews and recommendations
Previous update	2020	Continuously updated	2021
Structure	Recommendations followed by synopsis and supportive evidence text	Each recommendation is followed by evidence directly	Concise recommendations followed by longer evidence text
Recommendations	Strength of recommendations classified as: Strong Moderate Weak Moderate – no benefit Strong – harm	Follows GRADE approach: Strong, Weak Good practice statements	Not reported
Quality of evidence	Level A = high quality evidence Level B – R = moderate quality evidence from randomized studies Lever B – NR = moderate quality evidence from quality non-randomized studies Level C – LD = randomized or non-randomized studies with limitations in the execution Level C – EO = expert opinion	GRADE approach: High Moderate Low Very low	Not reported

The ERC guidelines acknowledge that evidence relevant to periviable (defined as gestational age less than 25 + 0 weeks) preterm neonates is limited and advise that application of their guideline to this group should be done with caution. The ANZCOR and AHA/AAP guidelines do not make specific recommendations about the management of neonates at the margins of viability, but ANZCOR suggests that consultations with families, using prognostic scores may be used to guide decisions about resuscitation at these gestations. These approaches are consistent with the findings that periviable preterm neonates have been underrepresented in neonatal randomized trials.^21^

Gestational age thresholds are used throughout the guidelines to structure recommendations for several domains of neonatal resuscitation. Commonly used thresholds include 28, 32, and 34 weeks’ gestation, although their application varies between topics and guidelines in the AHA/AAP and ANZCOR guidelines. The ERC guideline explicitly aims to apply standardized gestational age cut-offs of 32 + 0 and 37 + 0 across recommendations where possible, aiming to improve the clarity of the guideline.

When interpreting the differences between guidelines, it is important to recognize that not all variations necessarily carry the same clinical implications. Broadly, the differences identified in this review can be considered in two main categories: (1) differences that may lead to different clinical actions or management decisions, and (2) differences related to the presentation, structure, or scope of the guidelines that are unlikely to result in differences in clinical care. The majority of the variations observed between the guidelines fall into the latter category. Furthermore, when comparing the guidelines, distinctions were made between formal recommendations, good practice statements, statements indicating insufficient evidence, and the absence of explicit guidance.

## Umbilical cord management

The ILCOR evidence summaries underlying these recommendations include multiple gestational age categories and clinical scenarios, which can make translation into concise clinical guidelines challenging. In practice, guidelines may simplify these recommendations to facilitate implementation in the delivery room, where rapid decision-making is required. As a result, some differences between guidelines may reflect pragmatic choices in how to operationalize complex evidence rather than a fundamental disagreement on the interpretation of the data underpinning the guidelines. In addition, many decisions regarding cord management arise in situations where delayed cord clamping is not considered feasible, further emphasizing the importance of practical considerations in guideline development.

All guidelines recommend delayed cord clamping of more than 60 s for vigorous late preterm and term neonates (Table 2). The ANZCOR guideline pertaining to preterm neonates differs in that it recommends delaying cord clamping for at least 30 s whereas AHA/AAP and ERC guidelines both recommend a minimum of 60 s in this population (Table 2). This represents an example of a difference that may lead to different clinical actions, as the recommended minimum duration of delayed cord clamping differs between guidelines for preterm neonates. The recommendations from AHA/AAP were moderate and strong, whereas ANZCOR made weak recommendations based on very low and low certainty evidence for term and preterm infants respectively. Recent paired individual patient meta-analyses found that longer delays beyond 60 s may confer even greater benefits.^22^, ^23^

**Table 2:** Recommendations regarding umbilical cord management.

		**AHA/AAP 2025**			**ANZCOR 2025**			**ERC 2025**
		**Statement**	**Strength**	**Quality of evidence**	**Statement**	**Strength of recommendation**	**Certainty of evidence**	**Statement**
Delayed cord clamping	Term	DCC 60 s or more can be beneficial compared to ICC	Moderate	B – randomized	For term and late preterm infants born at ≥34 weeks’ gestation who are vigorous or deemed not to require immediate resuscitation at birth, ANZCOR suggests DCC ≥60 s.	Weak	Very low	Newborn infants without need for support: facilitate at least 60 s of delayed cord clamping.
	Preterm	DCC 60 s or more is recommended compared to ICC	Strong	A	For infants born at less than 34 weeks’ gestational age who do not require immediate resuscitation after birth, ANZCOR suggests DCC ≥30 s.	Weak	Low	Newborn infants without need for support: facilitate at least 60 s of delayed cord clamping.

Cord milking	Term	For nonvigorous neonates (GW 35 or more) I-UCM may be reasonable compared to ICC	Weak	B – randomized	There is insufficient evidence to recommend milking of the intact or cut cord for term infants.	N/A	N/A	Consider intact cord milking as an alternative, but only if delayed cord clamping cannot be performed
		For term neonates the usefulness of I-UCM compared to DCC is uncertain	Weak	C – limited data				
	Preterm	For preterm neonates born 28 + 0 to 36 + 6 weeks who do not require resuscitation and in whom DCC is not possible, I-UCM may be reasonable	Weak	B – randomized	There is insufficient evidence to recommend milking of the intact or cut cord late preterm infants.	N/A	N/A	Consider intact cord milking as an alternative in infants ≥28 weeks, but only if delayed cord clamping cannot be performed
		For preterm neonates (<28 GW) I-UCM should not be performed	Strong harm	B – randomized	ANZCOR suggests against intact cord milking for infants born at less than 28 + 0 weeks’ gestational age.	Weak	Very low	Do not milk the cord in preterm infants <28 weeks.

Intact cord stabilization	Term	No recommendations	N/A	N/A	No recommendations	N/A	N/A	If stabilization with intact cord can be safely undertaken, longer delayed cord clamping is preferred, especially in infants <34 weeks.
	Preterm	No recommendations	N/A	N/A	No recommendations	N/A	N/A	

*Abbreviations:* DCC = Delayed cord clamping. ICC = Immediate cold clamping. ICS = Intact cord stabilization. I-UCM = intact umbilical cord milking.

The ERC guideline states that “when possible”, intact cord stabilization with clamping based on physiological parameters may be performed especially in preterm neonates aged less than 34 weeks of gestation. ANZCOR guidelines state that a physiology-based approach may have advantages over time-based clamping but there is insufficient evidence to draw strong conclusions. They suggest that for both term and preterm infants, the optimal timing of cord clamping in the compromised neonate remains uncertain. The AHA/AAP guideline did not comment on intact cord stabilization. A recent large trial from Europe did not show an increase in survival without major cerebral injury or necrotizing enterocolitis in preterm neonates with physiology based cord clamping.^24^ Two recent meta-analyses did not find evidence of improved outcomes with intact cord stabilization and assessed the evidence as low to very low certainty.^25^, ^26^

Cord milking is addressed in all three guidelines. The AHA/AAP and ERC guidelines consider only intact cord milking, whereas the ANZCOR guideline evaluates both intact and cut cord milking. The ANZCOR guideline states that there is insufficient evidence to recommend cord milking in term or late preterm infants. In contrast, both the AHA/AAP and ERC guidelines state that intact cord milking is feasible in term neonates and in preterm neonates with a gestational age of ≥28 + 0 weeks, although the AHA/AAP recommendation is qualified as weak. In extremely preterm infants (GW < 28 + 0), all three guidelines recommend against cord milking because of a potential increased risk of intraventricular hemorrhage. The AHA/AAP and ERC guidelines provide a strong recommendation against cord milking, whereas the ANZCOR recommendation is weak and based on very low-certainty evidence. The recommendations are based on the large scale well conducted randomized study by Katheria et al which was terminated early due to increased risk of intraventricular hemorrhage in infants allocated to cord milking.^27^ However there are three previous meta-analyses of randomized controlled trials that did not find an association between any umbilical cord management strategy and intraventricular hemorrhage.^23^, ^28^, ^29^ The certainty of evidence was rated from very low to low, and all three meta-analyses included the study by Katheria et al in their analyses.^23^

The ILCOR evidence summary suggests against cord milking in extremely preterm neonates and gives a weak recommendation based on low certainty evidence.^30^ The summary notes that the recommendation is mostly driven by the single study which was stopped early. A similar recommendation against the use of cord milking in extremely preterm neonates appeared in the recent update of American College of Obstetricians and Gynecologists.^31^

The guideline recommendations against cord milking were based predominantly on a single trial and may overestimate treatment effects, particularly when the trial is stopped early.^32^ Typically, higher certainty of evidence is usually supported by a greater number of trials and participants.^33^ The increased risk was detected in the comparison with delayed cord clamping. In many cases, cord milking is considered as an alternative to immediate cord clamping in scenarios where delayed cord clamping is not feasible. Meta-analyses do not consistently demonstrate an increased risk of intraventricular hemorrhage associated with cord milking compared with immediate clamping, and milking may confer benefits over immediate cord clamping in terms of other outcomes.

## Airway management

All three guidelines recommend against routine airway suctioning in the presence of meconium-stained amniotic fluid. The AHA/AAP classifies this as a moderate, no-benefit recommendation based on Level B randomized evidence, whereas ANZCOR makes a weak recommendation supported by low-certainty evidence. All three guidelines also state that airway suctioning may be considered if tracheal obstruction is suspected during ventilation, and recommend that any such suctioning be performed under direct visualization (Table 3). These latter recommendations are based on expert opinion or made as good-practice statements.

**Table 3:** Airway management recommendations.

		**AHA/AAP 2025**			**ANZCOR 2025**			**ERC 2025**
		**Statement**	**Strength**	**Quality of evidence**	**Statement**	**Strength of recommendation**	**Certainty of evidence**	**Statement**
Clearing the airway		Suctioning of the mouth and nose can be considered in neonates if ventilation is required and the airway appears obstructed	Moderate	C – expert opinion	N/A	N/A	N/A	Perform suction under direct vision
		Intubation and tracheal suction can be beneficial for neonates who have evidence of tracheal obstruction during ventilation	Moderate	C – expert opinion	In general, suction should not be used except when newborns show obvious signs of obstruction either to spontaneous breathing or to positive pressure, ventilation and it should be done briefly and with care. Pharyngeal suction may be required to visualize the vocal cords during intubation	Good practice statement	N/A	Consider physical airway obstruction if lung inflation is unsuccessful despite alternative airway opening techniques.
		Routine oral, nasal, oropharyngeal, or endotracheal suctioning of neonates is not recommended regardless of the fluid (clear or meconium stained)	Strong no benefit	B – randomized	For all newborns exposed to meconium-stained amniotic fluid, ANZCOR suggests against routine direct laryngoscopy immediately after birth, with or without tracheal suctioning.	Weak	Low	Do not routinely suction meconium or amniotic fluid from infant’s’ airways because it delays initiating ventilation.

Airway devices	Video laryngoscopy	Video laryngoscopy can be useful for neonatal endotracheal intubation	Moderate	B – randomized	N/A	N/A	N/A	Where resources and training allow, the ERC recommends using a VL to intubate newborn infants, especially in settings where less experienced staff are intubating. Direct laryngoscopy remains a reasonable option, and such a laryngoscope should be available as a backup device.
	Laryngeal mask airway	It is reasonable to use a laryngeal mask as an alternative to endotracheal intubation for neonates aged GW 34 + 0 or more for whom ventilation via face mask is unsuccessful.	Moderate	C – limited data	ANZCOR suggests that a supraglottic airway should be considered during resuscitation of the term and near-term newborn (>34 weeks, approximately 2000 g) if facemask ventilation is unsuccessful.	Weak	Low	Consider using an appropriate size supraglottic airway (SGA) device: When facemask ventilation is ineffective; When a more definitive airway is required as an alternative to tracheal intubation; Where tracheal intubation is not possible or deemed unsafe because of congenital abnormality, a lack of equipment, or a lack of skill; When chest compressions are performed.
		It may be reasonable to use a laryngeal mask as the primary interface to administer ventilation instead of facemask for neonates with 34 + 0 or more gestational weeks	Weak	C – limited data	The supraglottic airway may be considered as a primary alternative to a facemask for positive pressure ventilation among newborns weighing more than 2000 g or delivered ≥34 weeks’ gestation, although there is insufficient evidence to support its routine use in this setting.	N/A	N/A	Consider using an appropriate size supraglottic airway (SGA) device as an alternative for facemask ventilation if SGA size permits
	Naso/oropharyngeal devices	N/A	N/A	N/A	N/A	N/A	N/A	Consider nasopharyngeal or oropharyngeal airway devices, especially when facemask ventilation may be difficult (e.g. micrognathia).
		N/A	N/A	N/A	N/A	N/A	N/A	Use oropharyngeal airway devices with caution in infants <34 weeks. They might contribute to airway obstruction.

The use of video laryngoscopy is recommended in both the AHA/AAP and ERC guidelines (Table 3). AHA/AAP makes a moderate strength recommendation with B-level evidence from randomized studies. The ANZCOR guideline has not yet commented on the use of video laryngoscopy. Three recent systematic reviews on the topic have found that the use of video laryngoscopy improves the first attempt success rate in neonates, and the reported certainties of evidence have varied between low to moderate.^34^, ^35^, ^36^ The reported benefit was higher in urgent neonatal intubations, where a number needed to treat of six was reported in the latest meta-analysis.^36^ European Society of Anaesthesiology and Intensive Care and British Journal of Anaesthesia joint guidelines on airway management in neonates and infants gave a strong recommendation with moderate quality evidence to use video laryngoscopy over direct laryngoscopy, however the authorship did not include neonatologists.^37^

The use of a supraglottic airway is considered in all three guidelines as an alternative to endotracheal intubation during resuscitation when a more secure airway is needed or when face mask ventilation is unsuccessful in late preterm and term neonates. AHA/AAP makes a moderate recommendation based on C-level evidence with limited data, and ANZCOR gives a weak recommendation based on low certainty evidence (Table 3). ANZCOR and AHA/AAP guidelines limit the recommendation to neonates of 34 weeks’ or above, whereas the ERC stated that supraglottic airway may be used if an appropriately sized device is available. All three guidelines recommend that a supraglottic airway may be used instead of face mask ventilation as the primary ventilation interface. The strength of recommendation is weak in the AHA/AAP guideline, and ANZCOR state that the evidence was insufficient to apply GRADE recommendations (Table 3) use of a supraglottic airway has recently been the focus of research, as the technique has the benefits of being easier to place than an endotracheal tube, and providing a more secure airway than a face mask. A recent Cochrane review of supraglottic airway vs mask ventilation assessed the certainty of evidence as moderate and concluded that the use of supraglottic airway instead of mask ventilation reduced the need for endotracheal intubation, and neonatal intensive care unit admission.^38^ Despite these findings, the use of supraglottic airways has remained limited and a recent multinational observational study reported that a supraglottic airway was used in 0.1% of the neonates who needed respiratory support.^7^ The influence of these updated guidelines on rates of supraglottic airway usage is of considerable interest.

Finally, the ERC guideline also mentions the possible use of oropharyngeal and nasopharyngeal airways with caution as adjuncts during airway management, whereas these devices are not specifically discussed in the AHA/AAP and ANZCOR guidelines.

## Ventilation in the delivery room

All three guidelines suggest that the optimal timing for initiating ventilation is within 60 s of birth (Table 4). The AHA/AAP guideline recommends providing ventilation within 60 s after birth for neonates who are apnoeic, gasping, or persistently bradycardic, and classifies this as a strong recommendation based on non-randomized data. ANZCOR similarly advises starting positive pressure ventilation when the newborn fails to establish respirations and has a heart rate below 100 beats per minute, noting that continuous positive airway pressure may be considered if the heart rate is increasing. The ERC guideline also advises initiating positive pressure ventilation as soon as possible in apnoeic or ineffectively breathing newborns, ideally within the first 60 s of life.

**Table 4:** Initial ventilation in the delivery room recommendations.

	**AHA/AAP 2025**			**ANZCOR 2025**			**ERC 2025**
	**Statement**	**Strength**	**Quality of evidence**	**Statement**	**Strength of recommendation**	**Certainty of evidence**	**Statement**
When to initiate	Ventilation should be provided within 60 s after birth in neonates who are gasping or apnoeic or who are persistently bradycardic (heart rate less than 100/min) despite appropriate initial steps.	Strong	B – non-randomized	However, if in response, the term or preterm newborn fails to establish effective respirations and heart rate is below 100 beats per min by 1 min of age and not increasing, Continuous Positive Airway Pressure (CPAP) or positive pressure ventilation should be commenced.	Good practice statement	N/A	If apnoeic, gasping or not breathing effectively, start PPV as soon as possible to inflate the lungs – ideally within 60 s

PIP	Initial peak inflation pressures of 20–30 cm H_2_O are reasonable with adjustment of peak inflation pressures to provide effective ventilation	Moderate	C – limited data	For commencing intermittent positive pressure ventilation, the suggested initial pressures are 30 cm H_2_O for term newborns and 20–25 cm H_2_O for premature newborns.	Good practice statement	N/A	Infants <32 weeks: starting inflation pressure 25 cm H_2_O. Infants ≥32 weeks: starting inflation pressure 30 cm H_2_O.
	Excessive peak inflation pressure can results in high tidal volume, which is potentially harmful and should be avoided	Strong harm	C – limited data	N/A	N/A	N/A	N/A

PEEP	In neonates receiving ventilation, it may be reasonable to provide positive end-expiratory pressure (PEEP)	Low	C – limited data	ANZCOR suggests the use of PEEP (commencing at 5–8 cm H_2_O pressure) during resuscitation of newborns wherever appropriate equipment is available.	Weak	Very low	Start with CPAP at 6 cm H_2_O as initial breathing support in: Spontaneously breathing infants <32 weeks with respiratory distress Spontaneously breathing infants ≥32 weeks with respiratory distress requiring supplemental O_2_
	N/A	N/A	N/A	High levels of PEEP (>8 cm H_2_O) have the potential to reduce pulmonary blood flow and cause pneumothorax, and should be used with caution	Good practice statement	N/A	N/A

Rate	It is reasonable to provide ventilation at a rate of 30–60/min	Moderate	C – limited data	Subsequent ventilation should be provided at 40–60 inflations per minute	Good practice statement		Aim for a positive pressure ventilation rate of 30 ventilations min^−1^ with an inflation time of approximately 1 s.

Time	It is reasonable to initiate with an inflation time of 0.5–1 s	Moderate	C – limited data	For term or late preterm newborns, it is not possible to recommend any specific duration for initial inflations because there are no published comparative trials.	N/A	N/A	Give 5 inflations with an inflation time up to 2–3 s.
	In preterm neonates (<28GW) the routine use of sustained inflations (5 s or more) to initiate ventilation is potentially harmful and should not be performed.	Strong harm	B – randomized	ANZCOR suggests against routine use of an initial sustained inflation (> 5 s) in preterm infants but sustained inflations may be considered in individual clinical circumstances or in research settings.	Weak	Low	The ERC guidance aligns with ILCOR and recommends against sustained inflations >5 s in preterm infants.

T-piece	It can be beneficial to use a T-piece resuscitator instead of a self-inflating bag with or without a PEEP valve, for administering ventilation to neonates, particularly preterm.	Moderate	B – randomized	ANZCOR suggests using a T-piece device for delivery of Intermittent Positive Pressure Ventilation (IPPV) or Continuous Positive Airway Pressure (CPAP) during newborn resuscitation.	Weak	Very low	Where possible, use a T-piece resuscitator capable of providing either CPAP or positive pressure ventilation + PEEP when giving ventilatory support, especially in the preterm infant.

CPAP	For spontaneously breathing preterm neonates who require respiratory support immediately after birth it is reasonable to use CPAP rather intubation and mechanical ventilation	Moderate	A	For spontaneously breathing preterm newborns <32 weeks’ gestation who have signs of respiratory distress in the delivery room and require respiratory support, ANZCOR suggests commencing CPAP in the first minutes after birth rather than intubation and ventilation.	Weak	Moderate	ERC recommends starting CPAP at 6 cm H_2_O in spontaneously breathing preterm infants <32 weeks over tracheal intubation and mechanical ventilation
	For spontaneously breathing preterm infants who require respiratory support immediately after birth, the effectiveness of high flow nasal cannula compared to CPAP is not well-established	Weak	C – limited data	N/A	N/A	N/A	N/A
	The usefulness of CPAP for spontaneously breathing term and late preterm neonates (34GW or more) who are at risk of having respiratory distress immediately after birth is not well-established	Weak	C – limited data	For spontaneously breathing term newborns with respiratory distress, a trial of CPAP may be considered, although there are no randomized trials to support this recommendation.	Good practice statement	N/A	The ERC considers it reasonable to start CPAP at 6 cm H_2_O in newborn infants ≥32 weeks with respiratory distress needing supplemental O_2_

The recommended initial peak inflation pressures (PIP) vary between guidelines (Table 4). The AHA/AAP guideline states that an initial PIP in the range of 20–30 cm H_2_O is reasonable, with adjustments made to achieve effective ventilation; this is supported by moderate-level evidence. ANZCOR suggests pressures of 20–30 cm H_2_O for term newborns and 20–25 cm H_2_O for preterm infants when commencing intermittent positive pressure ventilation. The ERC guideline recommends starting pressures of 25 cm H_2_O in infants <32 weeks’ gestation and 30 cm H_2_O in those ≥32 weeks and adjusting the needed pressure based on clinical findings and observations. The lower inflating pressures for preterm neonates are recommended based on older studies, which reported that 20–25 cmH_2_0 was associated with adequate chest wall expansion.^39^ However, no clinical studies have compared different peak inflating pressures in term or preterm neonates. Differences in recommended initial inflation pressures may also influence clinical practice, as these thresholds guide the pressures used during positive pressure ventilation in the delivery room.

Recommendations for the use of positive end-expiratory pressure (PEEP) also varied. The AHA/AAP guideline notes that the use of PEEP may be reasonable in neonates receiving ventilation, though this is based on low-quality evidence. ANZCOR suggests the use of PEEP (5–8 cm H_2_O) during resuscitation when equipment is available, but classifies this as a weak recommendation based on very low certainty evidence. The ERC guideline advises initiating continuous positive airway pressure (CPAP) at 6 cm H_2_O as the primary mode of support for spontaneously breathing infants—both those <32 weeks with respiratory distress and those ≥32 weeks who require supplemental oxygen (Table 4).

The ERC guideline also notes that respiratory support may be delivered using nasal interfaces when appropriate equipment is available, reflecting increasing interest in nasal CPAP delivery during initial stabilization of spontaneously breathing neonates, while the AHA/AAP and ANZCOR did not comment the interface. A recent randomized Australian study compared face mask and nasal interface in the CPAP delivery and reported a higher proportion of infants were successfully stabilized without escalation to positive pressure ventilation in the nasal interface group (58%) than in the face mask group (39%). They concluded that it was reasonable to deliver PEEP via a nasal interface in spontaneously breathing neonates.^40^ Similar results were reported in the recent Cochrane review of interfaces used in neonatal resuscitation which found that use of a nasal interface may reduce the need for intubation in the delivery room.^41^

The suggested ventilation rate varied between 30 and 60 in the guidelines (Table 4). The inflation times for initial inflations varied between the recommendations. The AHA/AAP recommendation stated that it is reasonable to start with an inflation time of 0.5–1 s and the class of recommendation was moderate. The ERC guideline recommends that all neonates should be given 5 initial inflations where inflation time is up to 2–3 s. The ANZCOR guideline does not give any recommendation as there have been no randomized trials on the topic (Table 5). All three guidelines recommended against the use of initial sustained inflations of more than 5 s in preterm neonates. These differences likely reflect pragmatic decisions related to training and implementation rather than divergent interpretations of the available evidence, as current studies do not clearly demonstrate superiority of one ventilation approach over another.

**Table 5:** Recommendations regarding oxygen use during resuscitation.

		**AHA/AAP 2025**			**ANZCOR 2025**			**ERC 2025**
		**Statement**	**Strength**	**Quality of evidence**	**Statement**	**Strength of recommendation**	**Certainty of evidence**	**Statement**
Saturation monitoring	Term	A pulse oximeter should be placed as soon as neonate requires respiratory support or oxygen	Strong	C – limited data	Oximetry is recommended when the need for resuscitation is anticipated, when CPAP or positive pressure ventilation is used, when persistent cyanosis is suspected, or when supplemental oxygen is used.	Good practice statement	N/A	Use pulse oximetry and O_2-_blenders during resuscitation or stabilization in the delivery area and check O_2_ and saturations every 30 s.
	Preterm	A pulse oximeter should be placed as soon as neonate requires respiratory support or oxygen	Strong	C – limited data	Oximetry is recommended when the need for resuscitation is anticipated, when CPAP or positive pressure ventilation is used, when persistent cyanosis is suspected, or when supplemental oxygen is used.	Good practice statement	N/A	Use pulse oximetry and O_2-_blenders during resuscitation or stabilization in the delivery area and check O_2_ and saturations every 30 s.

Sp0_2_ targets (see Fig. 1)	Term	1 min 60–65 2 min 65–70 3 min 70–75 4 min 75–80 5 min 80–85 10 min 85–95	N/A	N/A	1 min 60–70 2 min 65–85 3 min 70–90 4 min 75–90 5 min 80–90 10 min 85–90	N/A	N/A	3 min 70–75 5 min 80–85 10 min 85–95
	Preterm	1 min 60–65 2 min 65–70 3 min 70–75 4 min 75–80 5 min 80–85 10 min 85–95	N/A	N/A	1 min 60–70 2 min 65–85 3 min 70–90 4 min 75–90 5 min 80–90 10 min 85–90	N/A	N/A	3 min 70–75 5 min 80–85 10 min 85–95

Initial oxygen	Term	Infants born 35GW or more it may be reasonable to start with initial oxygen of 21%	Weak	C – limited data	For term and near-term newborn infants ANZCOR suggests that air should be used initially with supplemental oxygen reserved for those whose saturations do not meet the lower end of the targets despite respiratory support.	Weak	Low	Start at 21% O_2_
	Preterm	32 + 0–34 + 6: it may be reasonable to start with oxygen of 21–30%	Weak	C – limited data	For preterm infants <35 weeks’ gestation ANZCOR suggests commencing resuscitation either using room air or blended air and oxygen up to an oxygen concentration of 30% rather than higher initial oxygen concentration	Weak	Very low	≥32 weeks needing respiratory support start at 21% O_2_
		<32 GW: It may be reasonable to start with initial oxygen of 30–100%	Weak	C – limited data				Infants <32 weeks: Start at ≥30% O_2_ and avoid SpO_2_ <80% and/or bradycardia at 5 min of age

All three guidelines promoted the use of T-piece resuscitator, and the class of recommendation was moderate based on moderate quality randomized studies in the AHA/AAP guideline and a weak recommendation based on very low certainty evidence in the ANZCOR guideline.

Recommendations for the use of CPAP in the delivery room varied between guidelines, although all suggested that CPAP should be the first line of support for spontaneously breathing neonates (Table 4). The AHA/AAP guideline states that for spontaneously breathing preterm neonates requiring respiratory support immediately after birth, it is reasonable to initiate CPAP rather than proceed directly to intubation and mechanical ventilation. This was based on evidence assessed as being of moderate quality. ANZCOR similarly recommends CPAP for spontaneously breathing preterm infants <32 weeks’ gestation who show signs of respiratory distress and suggests commencing CPAP in the first minutes after birth rather than intubation. This is considered a weak recommendation based on limited evidence. For term or late preterm infants with respiratory distress, ANZCOR notes that a trial of CPAP may be considered, although no comparative trials exist to support the recommendation. The ERC guideline recommends that spontaneously breathing infants <32 weeks’ gestation receive CPAP at 6 cm H_2_O as the initial mode of respiratory support rather than tracheal intubation. For infants ≥32 weeks who exhibit respiratory distress requiring supplemental oxygen, the ERC considers it reasonable to initiate CPAP at 6 cm H_2_O in the delivery room.

The use of high-flow nasal cannula (HFNC) in initial respiratory management at birth is addressed only in the AHA/AAP guideline, which states that its effectiveness compared with CPAP in spontaneously breathing preterm neonates has not been established. Meta-analyses published in 2022 comparing HFNC with CPAP as primary respiratory support in preterm neonates reported higher treatment failure rates with HFNC, while rates of mechanical ventilation and other clinical outcomes were similar.^42^ However, these studies largely included stable infants, randomized after NICU admission, limiting their applicability to the delivery-room setting. Similarly, another meta-analysis suggested comparable efficacy and fewer adverse events with HFNC in neonates born at ≥28 weeks’ gestation, but was also based predominantly on NICU data.^43^ HFNC was discussed in the 2022 European consensus guidelines on respiratory distress syndrome as an alternative to CPAP in selected cases, based on moderate-quality evidence.^44^ Given the lack of delivery-room–specific data, the absence of stronger HFNC recommendations in neonatal resuscitation guidelines appears appropriate.

## Oxygen

Saturation monitoring to guide oxygen therapy was recommended in all guidelines for both term and preterm infants (Table 5). The AHA/AAP guideline gave a strong recommendation to use pulse oximeter monitoring when respiratory support or supplemental oxygen is required. A similar good practice recommendation was made by ANZCOR. The ERC guideline also recommends the use of pulse oximetry and oxygen blenders during resuscitation or stabilization in the delivery area and suggests considering SpO_2_ monitoring, with or without ECG monitoring, at the time respiratory support is initiated.

**Fig. 1:**
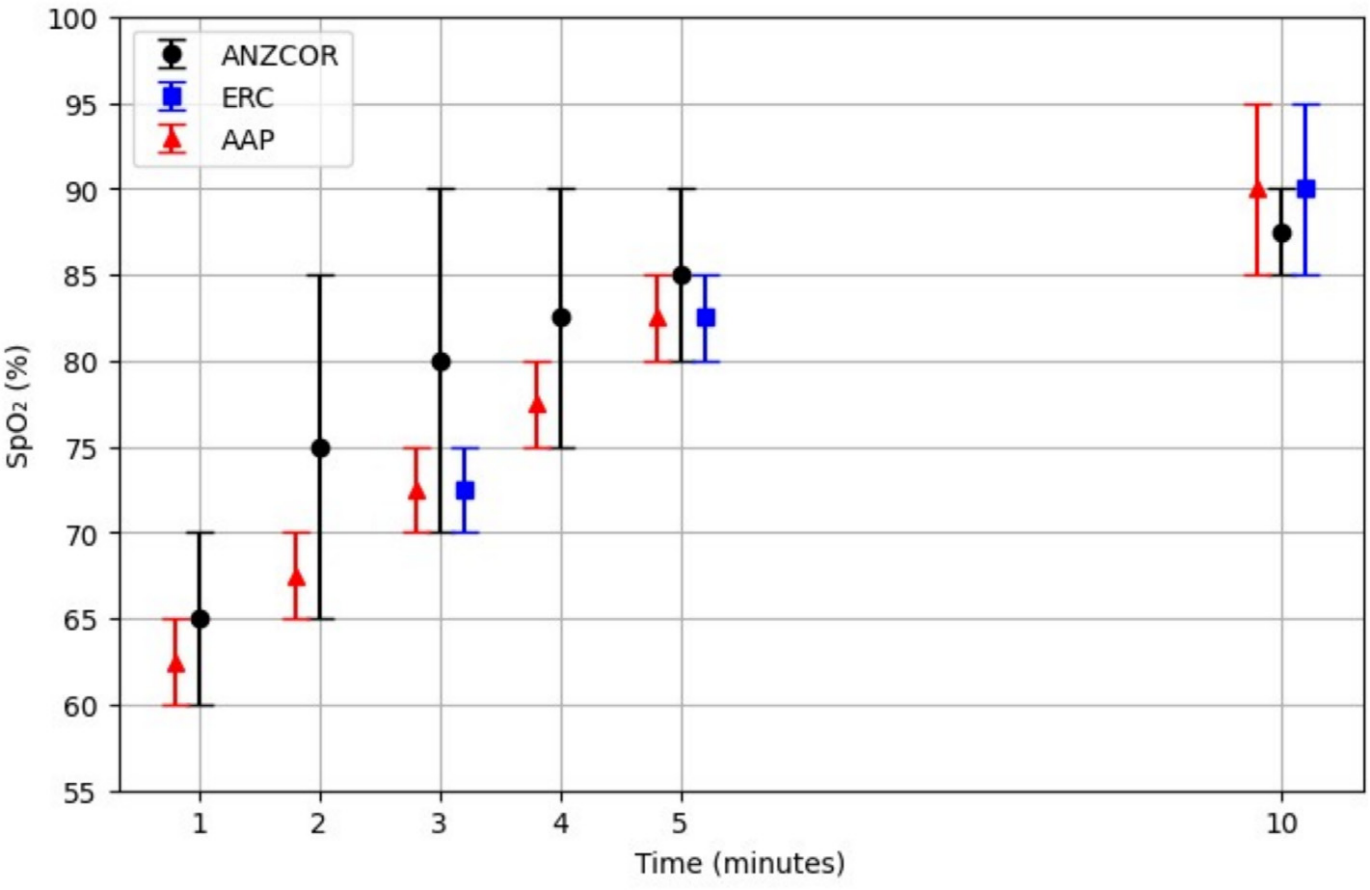
**Saturation targets in AHA/AAP, ANZCOR and ERC guidelines**.

Oxygen targets at specific time points were clearly presented in all guidelines (Fig. 1). There were some differences in the target saturation ranges, which were wider in the ANZCOR guidelines than those of the AHA/AAP and ERC (Fig. 1). The AHA/AAP guideline suggested that, based on expert opinion, targeting the preductal saturations of healthy neonates in the first minutes of life was an appropriate goal (Table 5).^45^, ^46^ Overall all the guidelines emphasized that supplemental oxygen should be titrated to achieve saturations in the target range.

All three guidelines suggested that the initial oxygen concentration should be 21% for term infants (Table 5). The three guidelines differed in their recommendations for preterm neonates. The AHA/AAP recommended starting with an oxygen concentration between 30% and 100% for neonates born with gestational age <32 + 0, and 21–30% for gestational weeks 32 + 0 to 34 + 6. In contrast, the ANZCOR guideline recommended initial oxygen concentration of 21–30% for all preterm neonates with gestational age less than 35 + 0. The ERC guidelines recommended initial oxygen concentration of 30–100% for all very preterm neonates with gestational age <32 + 0 and 21% for neonates of gestational age 32 + 0 or more (Table 5). These recommendations represent a difference that may lead to different clinical actions in the delivery room, as the suggested starting oxygen concentrations for preterm neonates vary substantially between the guidelines.

A recent individual patient network meta-analysis suggested that an initial oxygen concentration of 90% or more is associated with reduced mortality in preterm neonates with gestational weeks less than 32 + 0.^47^ The evidence was graded as very low to low certainty leading to a lack of confidence in the conclusions. One of the recent randomized studies published in 2025 on initial oxygen compared the compared the use of initial oxygen concentration of 100–30% in extremely preterm neonates during deferred cord clamping, and reported the target saturation of 80% or more at the age of 5 min was more likely to be achieved in the 100% group. However, as the study included only 140 neonates, it was underpowered to detect important differences in clinical outcomes.^48^ A large randomized study compared the initial oxygen of 30% to 60% with 1469 extremely preterm neonates and did not find evidence of a difference in rates of mortality or brain injury.^49^ Another recent review noted a lack of strong evidence, and recommended a strategy of starting with an initial oxygen concentration of 30% to 100% and then titrating by 10–20% every 30 s to achieve the target saturations.^50^ This is aligned with the current recommendation from AHA/AAP and ERC, whereas the ANZCOR recommendation is currently more conservative, suggesting commencement with an initial oxygen concentration of 21–30%. Pooled analysis of recently published and soon-to-be-completed trials may facilitate more consistent guidelines in future.

## Vascular access during resuscitation

Umbilical venous catheterization was promoted as the first choice in all three guidelines. The AHA/AAP guideline gave a strong recommendation based on C level evidence due to limited data (Table 6). The ANZCOR guideline assigned a good practice recommendation. The ERC guideline commented that clean, rather than sterile insertion may be appropriate in the emergency setting. A survey reported that a higher proportion of German neonatologists and other healthcare personnel attending neonatal resuscitations preferred umbilical vein catheterization as the first choice of vascular access.^51^ In a simulation study it took longer to place the umbilical vein catheter than to establish intraosseous access, but the success rates were comparable.^52^

**Table 6:** Vascular access during resuscitation.

	**AHA/AAP 2025**			**ANZCOR 2025**			**ERC 2025**
	**Statement**	**Strength**	**Quality of evidence**	**Statement**	**Strength of recommendation**	**Certainty of evidence**	**Statement**
Umbilical vein	Insertion of an umbilical vein catheter is recommended for newborn infants requiring emergency vascular access	Strong	C – limited data	An umbilical vein catheter (UVC) is the suggested intravascular route for adrenaline (epinephrine) and it can also be used for fluid administration. It can also be used for continued vascular access until an alternative route is established after admission to a neonatal unit.	Good practice statement	N/A	Use the umbilical vein for rapid emergency vascular access during resuscitation at birth. Perform emergency umbilical venous catheter (UVC) placement under clean rather than sterile conditions to ensure timely vascular access is secured. Consider the use of emergency umbilical venous catheter until some days after birth as it may still be achievable.
Intraosseous	Insertion of an intraosseous vascular access device can be useful newborn infants requiring vascular access if intravenous access is not successful or not feasible	Moderate	C – limited data	ANZCOR suggests this route can be used as an alternative, especially if umbilical or direct venous access is not available.	Weak	Very low	Use intraosseous (IO) access as an alternative method of emergency vascular access for medication and fluids. Consider device-specific weight limitations for IO related equipment. Ensure there is no extravasation when administering medication and fluids. Do not aspirate bone marrow; even when correctly positioned, it is often not possible.
Peripheral vein	N/A	N/A	N/A	Inserting a peripheral venous cannula can be very difficult in a shocked newborn and can take too long.	N/A	N/A	N/A

Intraosseous vascular access was mentioned in all three guidelines, and all recommended consideration of the intraosseous route if umbilical vein catheterization was not appropriate or achievable (Table 6). The AHA/AAP made a moderate recommendation based on C level evidence with limited data. ANZCOR made a weak recommendation based on very low certainty evidence. The ERC guideline stated that bone marrow aspiration should not be attempted, as it may not be informative even when the device is correctly positioned (Table 6). The ERC guideline also acknowledges that complications and adverse events have been reported for both umbilical venous catheterization and intraosseous access, whereas the other guidelines provide less detailed discussion of these potential complications. Furthermore, the guidelines did not comment on the best device for intraosseous access. Previous studies on this topic are limited, but a single study performed on stillborn neonates found the best success rate occurred using a butterfly needle, although the success rate was only around 60% with that technique.^53^ Peripheral vein access was commented in the ANZCOR guideline, which suggested that insertion in a shocked neonates is difficult and time consuming. The ERC guideline states that there have been no studies conducted on peripheral access in infants requiring resuscitation at birth.

In general, there was consistency in the guidelines regarding vascular access routes in neonatal resuscitation.

## Medications and fluids in resuscitation

The indication for epinephrine use was the same in all three guidelines. Epinephrine should be given when the heart rate remains less than 60 per minute with adequate ventilation and chest compressions (Table 7). The class of recommendation was strong with moderate non-randomized evidence in the AHA/AAP guideline, whereas the ANZCOR made a weak recommendation based on very low certainty evidence. The recommended initial dose was the same in all, and AHA/AAP and ANZOCR recommended repetition of epinephrine every 3–5 min. The ERC guideline suggested repetition every 4 min, which aligns with the pediatric and adult life support algorithms.

**Table 7:** Medications and fluids used during resuscitation.

		**AHA/AAP 2025**			**ANZCOR 2025**			**ERC 2025**
		**Statement**	**Strength**	**Quality of evidence**	**Statement**	**Strength of recommendation**	**Certainty of evidence**	**Statement**
Epinephrine	Indication	In neonates whose heart rate has not increased to 60/min or more after optimizing ventilation and chest compressions, it is recommended to administer intravascular epinephrine	Strong	B – non-randomized	ANZCOR suggest that if the heart rate has not increased to 60 beats per minute or greater after optimizing ventilation and chest compressions, then intravascular adrenaline (epinephrine) should be given as soon as possible.	Weak	Very Low	Resuscitation medication may be considered where, despite adequate control of the airway, effective ventilation, and chest compression for at least 30 s, HR remains <60 min^−1^ and is not increasing.
	Dose	The recommended dose to administer epinephrine is 0.01–0.03 mg/kg intravenous	Strong	B – non-randomized	The suggested intravenous dose is 10–30 µg/kg (0.1–0.3 mL/kg of a 1:10,000 solution) by a quick push	Weak	Very Low	10–30 µg kg^−1^ (0.1–0.3 mL kg^−1^ of 1:10,000 adrenaline [0.1 mg/mL])
	Repeating	It may be reasonable to administer additional doses of epinephrine ever 3-5minuts intravascularly, if the heart rate remains less than 60/min	Weak	C – limited data	This dose can be repeated every 3–5 min if the heart rate remains <60 beats per minute despite effective ventilation and cardiac compressions.	Weak	Very Low	Give subsequent doses every 4 min if HR remains <60 min
	Intratracheal	It is reasonable to administer intratracheal epinephrine at a larger dose (0.05–0.1 mg/kg) while vascular access is being obtained	Moderate	C – limited data	There is insufficient evidence for the use of endotracheal adrenaline (epinephrine), but it is likely that a higher dose will be required to achieve similar blood levels and effect. ANZCOR suggests that if the tracheal route is used, doses of 50–100 µg/kg (0.5–1 mL/kg of a 1:10,000 solution) should be given.	Weak	Very Low	Give intratracheal adrenaline at dose of 100 µg kg^−1^ (1 mL kg^−1^ of 1:10,000 adrenaline [0.1 mg/mL])
		In neonates who do not have an adequate response to an intratracheal dose of epinephrine, it is reasonable to administer intravascular epinephrine as soon as access is obtained regardless of the dosing interval	Moderate	B – non-randomized	N/A	N/A	N/A	If HR remains <60 min^−1^: as soon as umbilical venous catheter/ IO access is obtained, immediately give a dose via this route, irrespective of when the intra-tracheal dose was given

Volume replacement	Indication	It may be reasonable to administer a volume expander to neonate with evidence of hypovolemia, based on history, physical examination, who remain bradycardic despite ventilation, chest compressions, and epinephrine	Weak	C – expert opinion	Intravascular fluids should be considered when there is suspected blood loss, the newborn appears to be in shock (pale, poor perfusion, weak pulse) and has not responded adequately to other resuscitative measures. Isotonic crystalloid (e.g. 0.9% sodium chloride or Hartmann’s solution) should be used in the first instance, but may need to be followed with red cells and other blood products suitable for emergency transfusion, in the setting of critical blood loss.	Good practice statement	N/A	Give 10 mL/kg of group O Rh-negative blood or isotonic crystalloid solution if suspected blood loss or in a newborn infant unresponsive to other resuscitative measures
	Dose	It may be reasonable to use normal saline (0.9% sodium chloride) or blood at 10–20 ml/kg for volume expansion	Weak	C – expert opinion	The initial dose is 10 mL/kg given by IV push (over several minutes). This dose may be repeated after observation of the response.	Good practice statement	N/A	10 mL/kg of group O Rh-negative blood or isotonic crystalloid solution

Glucose		N/A	N/A	N/A	N/A	N/A	N/A	If blood glucose is low: give glucose 200 mg kg^−1^ (2 mL/kg of 10% glucose).

Intratracheal administration was mentioned by all three guidelines. AHA/AAP made a moderate recommendation based on weak limited data that intratracheal epinephrine could be used in a larger dose of 0.05–0.1 mg/kg while vascular access is being obtained. When vascular access is achieved epinephrine should be immediately repeated if still needed, a moderate recommendation based on moderate quality non-randomized evidence (Table 7). The ANZCOR guideline stated that the evidence is insufficient, but if intratracheal administration is used, the dose should be 0.05–0.1 mg/kg, and gave a weak recommendation based on very low certainty evidence. It made no statement regarding the repetition of epinephrine once intravascular access was established. The ERC guideline stated that intratracheal epinephrine dose should be 0.1 mg/kg and that if there is no heart rate response, epinephrine should be immediately repeated once vascular access has been established (Table 7).

All guidelines acknowledged the limited evidence regarding the role of volume replacement in neonatal resuscitation. All recommended the use of isotonic crystalloid solution with 10 ml/kg initial dosing while monitoring the response. Another option recommended was to administer blood if suspected blood loss (Table 7). These were based on expert opinions.

Glucose use was only commented in the ERC guideline, where it was stated that if blood glucose is low during the resuscitation, a bolus of 10% glucose of 2 ml per kilogram should be administered (Table 7).

The ERC guideline removed mention of sodium bicarbonate and naloxone in this update. This brought it into line with both ANZCOR and AHA/AAP, which have not included recommendations on these medications in recent guidelines. Despite a long history of use of both agents in neonatal resuscitation, evidence regarding these two interventions remains insufficient, and current guidelines suggest that the potential harms are likely to exceed the potential benefits.^54^ A recent update to Pediatric Pharmacy Association gave a strong recommendation against the use of naloxone in neonates due to increased seizure risk.^55^

## Conclusion

Although the AHA/AAP, ANZCOR, and ERC neonatal resuscitation guidelines are all derived from the same ILCOR evidence summaries, important differences exist in their structure, transparency, and specific clinical recommendations. Variability was evident across multiple domains, including umbilical cord management, airway strategies, ventilation parameters, oxygen supplementation, and the use of adjunctive devices and medications. In several instances, strong recommendations were issued despite low or very low certainty evidence. Where high-quality evidence is lacking, differences between guidelines often reflect pragmatic choices regarding clinical thresholds rather than fundamental disagreement regarding the underlying evidence. Some variation between guidelines may appropriately reflect differences in healthcare systems, resource availability, device availability, and training practices. However, greater harmonization may be beneficial when differences arise primarily from varying interpretations of the same underlying evidence base. Importantly, the lack of high-quality randomized trials, particularly involving extremely preterm and periviable neonates remains a major limitation.

## CRediT authorship contribution statement

**Ilari Kuitunen:** Writing – original draft, Visualization, Methodology, Investigation, Formal analysis, Data curation, Conceptualization. **Peter G. Davis:** Writing – review & editing, Validation, Supervision, Methodology, Conceptualization.

## Declaration of competing interest

The authors declare that they have no known competing financial interests or personal relationships that could have appeared to influence the work reported in this paper.
